# Moderate-intensity treadmill exercise improved the anticonvulsant efficacy of topiramate and attenuated anxiety and depressive-like side effects in epileptic rats by upregulating 5-HT1A receptors in the hippocampus and cerebral cortex

**DOI:** 10.1016/j.ibneur.2026.08.006

**Published:** 2026-08-09

**Authors:** Ghazaleh Goudarzi, Babak Aliyari, Mansoureh Soleimani, Gelareh Vahabzadeh, Homa Rasoolijazi, Fariba Karimzadeh

**Affiliations:** a Cellular and Molecular Research Center, Iran University of Medical Sciences, Tehran, Iran; b Department of Anatomy, School of Medicine, Iran University of Medical Sciences, Tehran, Iran; c Student research committee, Iran University of Medical Sciences, Tehran, Iran; d Razi Drug Research Center, Iran University of Medical Sciences, Tehran, Iran; e Department of Pharmacology, School of Medicine, Iran University of Medical Sciences, Tehran, Iran

**Keywords:** Epilepsy, 5-HT1A serotonin receptor, Pentylenetetrazol, Topiramate, Exercise

## Abstract

**Background:**

Topiramate (TPM) is a broad-spectrum antiseizure medication associated with dose-limited mood-related adverse effects, including depression, which significantly impair patient quality of life and treatment adherence. Although physical exercise confers neuroprotective and mood-stabilizing benefits, its potential to counteract TPM-induced behavioral deficits and the underlying neurobiological mechanisms, particularly within the serotonergic system, remains largely unexplored.

**Objective:**

The present study investigated whether exercise reduced the effective dose of topiramate and attenuates its depressive-like side effects in epileptic rats.

**Methods:**

Male Wistar rats were randomly assigned to nine groups (n = 10/group): Sham, Seizure, Exercise (EX), TPM (25 mg), TPM (50 mg), TPM (70 mg), EX + TPM (25 mg), EX + TPM (50 mg), and EX + TPM (70 mg).

The exercise protocol consisted of treadmill running (30 min/day, 5 days/week for 4 weeks). Seizure severity and latency were assessed using the Racine scale. Depressive-like behaviors and anxiety were evaluated by the Tail Suspension Test (TST) and the Elevated Plus Maze (EPM). Immunohistochemistry was performed to quantify 5-HT1A receptor immunoreactive staining intensity in the hippocampal CA1, CA3, and cerebral cortex.

**Results:**

Combination therapy (EX+TPM (50 mg) and EX + TPM (70 mg)) demonstrated superior anticonvulsant effects, significantly reducing seizure scores and prolonging seizure latency compared to TPM monotherapy (*p < 0.001*). TPM monotherapy dose-dependently exacerbated depressive-like behaviors (e.g., increased immobility in the TPM (70 mg) group, *p < 0.001*). Crucially, exercise attenuated these adverse effects in all EX + TPM groups. 5-HT1A receptor expression was significantly downregulated in the Seizure and TPM (25 mg) groups compared with the Sham and Exercise groups. In contrast, the EX + TPM (50 mg) and EX + TPM (70 mg) groups showed significant upregulation of 5-HT1A receptors in the hippocampus and cerebral cortex (*p < 0.001*).

**Conclusion:**

The current findings demonstrate that adjunctive moderate-intensity treadmill exercise potentiates the anticonvulsant efficacy of topiramate and effectively counteracts its depressive-like side effects and anxiety. This behavioral improvement was correlated with exercise-mediated upregulation of 5-HT1A receptors, suggesting a potential mechanistic link. These findings provide a preclinical rationale for further investigation into the potential benefits of exercise as an adjunctive therapy in epilepsy, though clinical translation requires additional studies.

## 1 Introduction

Epilepsy is a chronic neurological disorder characterized by sustained disruptions in neuronal excitability, leading to spontaneous and recurrent seizures that reflect abnormal, synchronized neural circuit activity (Du et al., 2026). These epileptiform discharges impair physiological communication between the cerebral cortex and subcortical regions, resulting in transient impairments in behavior, motor function, and cognition (Dahal et al., 2019). Current clinical guidelines establish pharmacotherapy as the principal intervention for epilepsy, with the primary objective of achieving seizure suppression (Tomson et al., 2023). A fundamental mechanism of action for many antiseizure drugs involves restoring neurochemical equilibrium by enhancing GABA-mediated inhibition and/or reducing glutamatergic excitation (Sears and Hewett, 2021). Topiramate, a broad-spectrum antiseizure medication, modulates neuronal excitability through three primary mechanisms: blockade of voltage-gated sodium and calcium channels, and enhancement of GABAergic activity (Bai et al., 2022). Despite its favorable pharmacodynamic profile, mood disturbances, including depression and suicidal ideation, have been reported as adverse effects. These side effects are primarily attributed to excessive inhibition of glutamatergic neurotransmission, specifically via blockade of AMPA and kainate receptors. Additionally, topiramate may contribute to mild central nervous system metabolic acidosis through the inhibition of carbonic anhydrase. It has been proposed that the drug may indirectly elicit depressive-like symptoms by modulating dopaminergic and serotonergic pathways; however, the precise mechanisms underlying these effects remained to be fully elucidated (Hudon and Proulx, 2022, Soleimani Meigoni et al., 2022).

Physical exercise is increasingly recognized as a non-pharmacological strategy that confers neuroprotective and mood-stabilizing benefits in individuals with epilepsy (Tero-Vescan et al., 2025). Aerobic exercise enhances serotonergic neurotransmission through multiple pathways, including increased tryptophan bioavailability, elevated serotonin synthesis, and adaptive changes in receptor expression. These changes include region-specific modulation of 5-HT1A receptors, downregulation of inhibitory somatodendritic autoreceptors in the raphe nuclei, and upregulation of excitatory postsynaptic receptors in target regions such as the hippocampus and cerebral cortex (Heijnen et al., 2016, Sha et al., 2026).

Accumulating evidence from preclinical and clinical studies underscores the role of regular physical activity in modulating neuronal excitability and conferring seizure protection. The underlying mechanisms are multifactorial and include enhanced neuroplasticity, facilitated by the upregulation of brain-derived neurotrophic factor (BDNF). This neuroplastic adaptation is accompanied by reinforcement of inhibitory GABAergic neurotransmission and stabilization of synaptic homeostasis (Ebrahimnezhad et al., 2023, Ambrogini et al., 2023).

Mood disorders, particularly depression, are highly prevalent among patients with epilepsy. Physical exercise exerts profound therapeutic effects on mood regulation and affective disorders. Its efficacy in ameliorating depressive-like and anxiety-related behaviors is mediated through several interconnected neurobiological pathways. Exercise modulates monoaminergic systems, including serotonin, norepinephrine, and dopamine (Pietrelli et al., 2018, Zhang et al., 2022, Albert et al., 1999). Regular physical activity attenuates neuroinflammation by downregulating pro-inflammatory cytokines such as IL-1β and TNF-α, a mechanism linked to the pathophysiology of depression (Xiao et al., 2021, Brás et al., 2022). Aerobic exercise promotes hippocampal neurogenesis, thereby counteracting the hippocampal atrophy frequently observed in mood disorders (Vints et al., 2024).

Despite the advent of newer-generation antiseizure medications, topiramate remains an indispensable therapeutic agent for a distinct subset of patients with drug-resistant epilepsy (Deng et al., 2025).

Clinical evidence and pharmacogenetic insights suggested that, for certain individuals, topiramate is uniquely capable of establishing effective seizure control. Furthermore, inter-individual variability in pharmacokinetics, pharmacodynamics, and polymorphisms affecting drug targets and neural circuits may render topiramate the most effective option for a patient’s specific seizure fingerprint. Consequently, in contemporary epilepsy management, topiramate is often regarded as an essential rescue therapy for patients who have proven refractory to multiple first and second-line treatment regimens. Nevertheless, despite its well-established antiseizure efficacy, the clinical utility of topiramate is limited by neuropsychiatric adverse effects, most notably depression and suicidal ideation.

Despite substantial evidence supporting the beneficial effects of exercise on brain function and its anti-inflammatory properties, as well as the well-documented side effects of topiramate, no study has yet investigated whether exercise can mitigate the adverse psychiatric effects of topiramate while maintaining or enhancing its anticonvulsant efficacy. Specifically, the role of 5-HT1A receptor modulation in this interaction has remained unexplored. Given the high comorbidity of depression and epilepsy, identifying a non-pharmacological strategy to improve the therapeutic profile of AEDs could have significant clinical implications.

Therefore, the present study was designed to address the following research question: Does moderate-intensity aerobic exercise enhance the anticonvulsant efficacy of topiramate while attenuating its depressive-like side effects in a PTZ-induced seizure model, and if so, is this effect associated with changes in 5-HT1A receptor expression?

We hypothesized that exercise would reduce the effective dose of topiramate and prevent the reduction of 5-HT1A receptor expression, thereby improving both seizure control and mood-related outcomes. To test this hypothesis, we investigated the effect of moderate-intensity treadmill exercise on the anticonvulsant efficacy of topiramate as well as its depressive-like side effects in a rat model of PTZ-kindling. In addition, the 5-HT1A receptor expression in the hippocampus and cerebral cortex as the possible involved mechanism was assessed.

## 2 Methods

### 2.1 Animals

Male Wistar rats (250–300 g) were housed under controlled environmental conditions (22 ± 2 °C, 12 h light/dark cycle) and allowed to habituate to the laboratory for one week prior to the start of the experiment. All experimental procedures were conducted in accordance with the guidelines approved by the Animal Ethics Committee of Iran University of Medical Sciences, Tehran, Iran (Ethics code: IR.IUMS.AEC.1402.111). The animals were randomly divided into nine groups (n = 10 per group) as follows:

Sham group: Rats received normal saline (0.9% NaCl) administered intraperitoneally every other day for four weeks. The use of this physiologically inert vehicle ensured that any observed effects in the treatment groups could be specifically attributed to the pharmacological action of the test compound, thereby controlling for solvent-related confounding variables as per established experimental design principles (Barzroodi Pour et al., 2021).

Seizure group: Animals received pentylenetetrazol (PTZ, i.p.) according to the same schedule used for the Sham group to induce seizure activity.

Exercise group (EX): Rats performed treadmill running according to the exercise protocol, consisting of 30-minute sessions, five days per week, for a total duration of four weeks.

TPM (25 mg), TPM (50 mg), and TPM (70 mg) groups: Topiramate was injected i.p. at doses of 25, 50, and 70 mg/kg, respectively. Thirty minutes after topiramate administration, seizures were induced by injection of PTZ following the same protocol as used in the Seizure group.

EX + TPM (25 mg), EX + TPM (50 mg), and EX + TPM (70 mg) groups: Animals were forced to run on the treadmill. Five hours after exercise, TPM was administered intraperitoneally at doses of 25, 50, or 70 mg/kg, and seizure induction with PTZ was carried out 30 min later.

### 2.2 Assessment of convulsive behaviors

After each PTZ (35 mg/kg; Sigma Aldrich, Germany) injection, convulsive behaviors were continuously recorded for 30 min using a high-resolution video camera. Two independent observers, blinded to the treatment groups and trained in seizure behavior scoring, analyzed the recordings.

Seizure severity was assessed using the Racine scale with stages from 0 to 6, as follows:

0 = no seizure; 1 = immobility; 2 = body rigidity; 3 = repeated scratching, impaired balance, and rapid head movements; 4 = forelimb clonus with rearing and falling; 5 = repeated occurrence of stage 4 behaviors, and 6 = severe tonic-clonic seizures.

Latency was defined as the elapsed time from PTZ injection to the onset of the first discernible seizure behavior (Karimzadeh et al., 2013).

### 2.3 Exercise protocol

Treadmill running was performed for 30 min, five days per week, in the exercise groups. Treadmill speed was gradually increased by 5 m/min every five minutes until reaching a maximum of 25 m/min. The treadmill was set at a 0° incline throughout the exercise period. Each session began with a three-minute warm-up at 8 m/min and concluded with a three-minute cool-down at the same speed.

### 2.4 Behavioral assessments

#### 2.4.1 Tail Suspension Test

The Tail Suspension Test (TST) was conducted in all experimental groups to evaluate depression-like behavior and behavioral despair. Rats were suspended by the tail in a chamber designed to prevent escape or contact with surrounding surfaces. Immobility duration was recorded over six minutes; the first two minutes were excluded to avoid initial escape attempts. The primary analysis was focused on the last four minutes. Rats spending more than 30% of the time climbing their tails or exhibiting signs of severe stress were excluded and replaced (Enriquez, 2023).

#### 2.4.2 Elevated plus maze test

The elevated plus maze (EPM) was used to evaluate anxiety-like behavior, which is frequently comorbid with depressive-like states. While the EPM does not directly measure depression, it provides complementary information about the emotional status of the animals. The tail suspension test (TST) was specifically used to assess depressive-like behavior.

Each rat was placed individually at the center of the Elevated Plus Maze (EPM) and allowed to explore freely for five minutes. The total time spent as well as the number of entries into the open and closed arms were recorded by a ceiling-mounted camera system. In depression-like states, rodents generally showed decreased exploratory behavior toward open arms and increased preference for enclosed arms, reflecting diminished motivation and behavioral despair linked to depressive phenotypes (Xia et al., 2023).

### 2.5 Immunohistochemistry analysis

Immunohistochemical analysis was performed to quantify 5-HT1A receptor immunoreactive staining intensity within the CA1 and CA3 subfields of the hippocampus and the cerebral cortex. Following deep anesthesia with chloral hydrate (350 mg/kg; Sigma–Aldrich), animals were transcardially perfused with 250 ml of saline, followed by 400 ml of 4% paraformaldehyde (PFA) in 0.1 M phosphate buffer. Brains were then carefully dissected, post-fixed in the same 4% PFA solution for one week at 4 °C, and subsequently processed for paraffin embedding.

Serial coronal sections (7 µm thickness) were obtained from regions corresponding to approximately 1.8–3.3 mm posterior to the bregma using a rotary microtome. Three sections per brain, spaced at 30 µm intervals, were deparaffinized in xylene and rehydrated through a graded ethanol series. After thorough rinsing with phosphate-buffered saline (PBS, 0.1 M, pH 7.4), heat-induced antigen retrieval was performed by incubating the sections in Tris-EDTA buffer (pH 9.0) at 95 °C for 20 min. Following a 20-minute cool-down period at room temperature and subsequent PBS washes, non-specific binding sites were blocked using a solution of 1% bovine serum albumin (BSA) in PBS for 20 min.

Sections were then incubated overnight at 4 °C with rabbit polyclonal anti-rat 5-HT1A receptor antibody (orb10007, diluted 1:100). After being thoroughly rinsed in PBS, the sections were treated with a FITC-conjugated goat anti-rabbit IgG secondary antibody (diluted 1:500) for one hour at room temperature. Finally, sections were washed, mounted, and coverslipped for microscopic analysis.

Quantitative assessment of 5-HT1A receptor immunoreactive staining intensity in the CA1 and CA3 subfields of the hippocampus and the cerebral cortex was performed using Infinity analysis software (version 4.6) across standardized 1 mm² microscopic fields. The staining intensity was expressed as arbitrary units (A.U.).

### 2.6 Statistical analysis

All quantitative data were presented as mean ± S.E.M Normality of data distribution was assessed for each group separately using the Kolmogorov-Smirnov test (n = 10 per group). As the data met the assumptions of normality, one-way analysis of variance ANOVA was performed, and Scheffe’s post-hoc test was used to correct for multiple comparisons, thereby controlling the family-wise error rate. All pairwise comparisons were adjusted using this method to minimize the risk of Type I errors.

The analyses were performed with IBM SPSS Statistics version 27.0.1 (IBM Corp., Armonk, NY, USA). Each experimental group consisted of ten rats, and statistical significance was accepted at *p* < 0.05.

## 3 Results

### 3.1 Score of convulsive behaviors

**Fig. 1:**
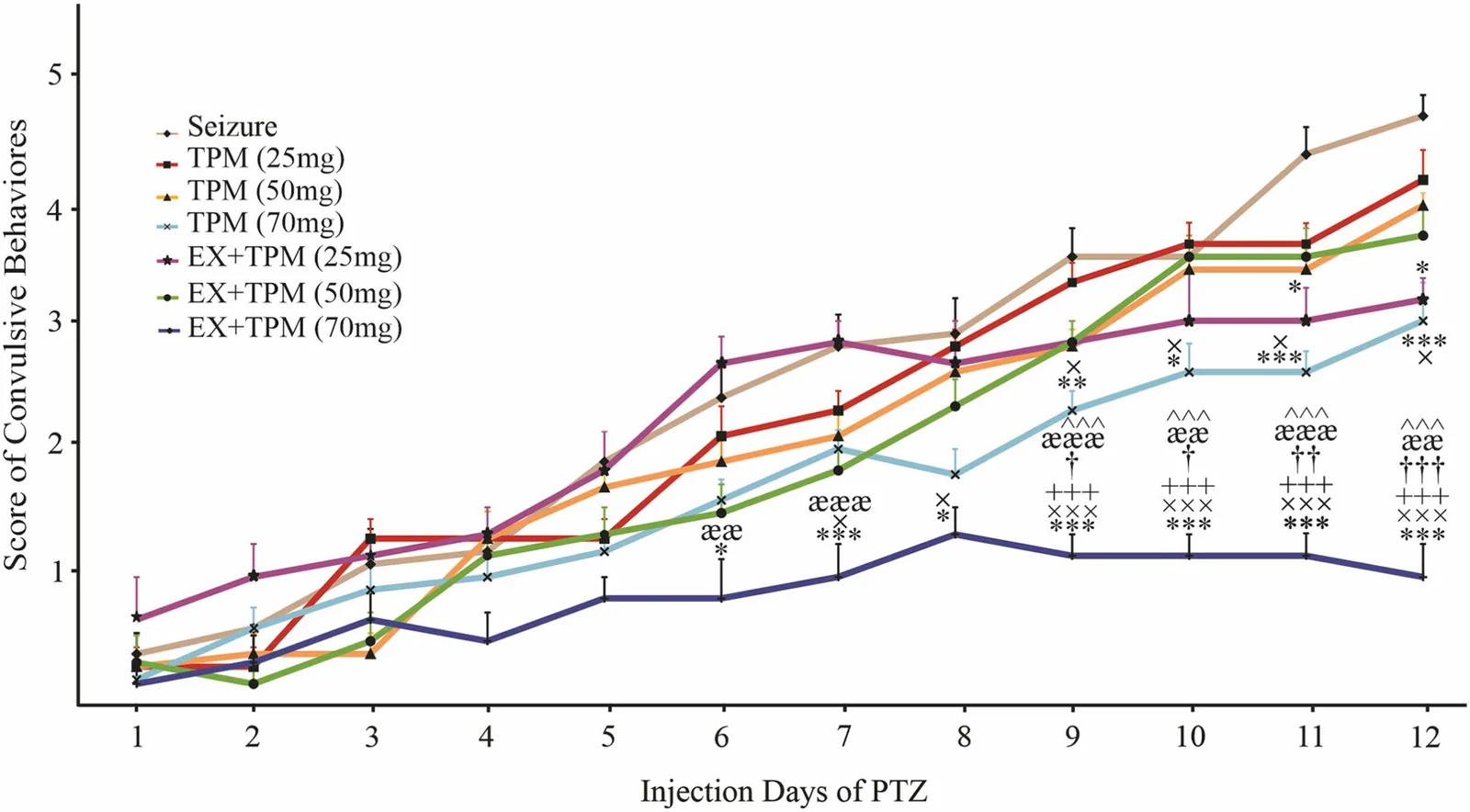
Seizure behavior scores: Temporal profile of seizure severity scores was assessed using the Racine scale. Data are presented as mean ± S.E.M. (error bars). Normality of distribution was confirmed using the Kolmogorov-Smirnov test, and individual data points are shown to illustrate variability. The combination of exercise and topiramate (EX+TPM) resulted in significantly lower seizure scores than those observed in both the Seizure group and the groups receiving topiramate alone (at doses of 25, 50, and 70 mg). The symbols *, × , + , †, æ, ^ and £ indicate a significant difference in comparison with the Seizure, TPM (25 mg), TPM (50 mg), TPM (70 mg), EX + TPM (25 mg), and EX + TPM (50 mg) and EX + TPM (70 mg) groups, respectively. One, two, or three repetitions of symbols indicate *p < 0.05*, *p < 0.01*, and *p < 0.001*, respectively.

Seizure behavior scores are presented in Table 1 and Fig. 1.

**Table 1:** Mean ± S.E.M. of seizure behavior scores.

Injections	Groups						
	Seizure	TPM (25 mg)	TPM (50 mg)	TPM (70 mg)	EX + TPM (25 mg)	EX + TPM (50 mg)	EX + TPM (70 mg)
1	0.4 ± 0.16	0.3 ± 0.15	0.3 ± 0.15	0.2 ± 0.13	0.66 ± 0.33	0.33 ± 0.21	0.16 ± 0.16
2	0.6 ± 0.16	0.3 ± 0.15	0.4 ± 0.16	0.6 ± 0.16	1.0 ± 0.25	0.16 ± 0.16	0.33 ± 0.21
3	1.1 ± 0.27	1.3 ± 0.15	0.4 ± 0.16	0.9 ± 0.23	1.16 ± 0.16	0.5 ± 0.22	0.66 ± 0.21
4	1.2 ± 0.13	1.3 ± 0.21	1.3 ± 0.21	1.0 ± 0.14	1.33 ± 0.21	1.16 ± 0.16	0.5 ± 0.22
5	1.9 ± 0.23	1.3 ± 0.15	1.7 ± 0.15	1.2 ± 0.13	1.83 ± 0.3	1.33 ± 0.21	0.83 ± 0.16
6	2.4 ± 0.26	2.1 ± 0.23	1.9 ± 0.17	1.6 ± 0.16	2.66 ± 0.21	1.5 ± 0.22	0.83 ± 0.3
7	2.8 ± 0.24	2.3 ± 0.15	2.1 ± 0.17	2.0 ± 0.14	2.83 ± 0.16	1.83 ± 0.16	1.0 ± 0.25
8	2.9 ± 0.27	2.8 ± 0.13	2.6 ± 0.26	1.8 ± 0.2	2.66 ± 0.33	2.33 ± 0.21	1.33 ± 0.21
9	3.5 ± 0.22	3.3 ± 0.15	2.8 ± 0.13	2.3 ± 0.15	2.83 ± 0.16	2.83 ± 0.16	1.16 ± 0.16
10	3.5 ± 0.16	3.6 ± 0.16	3.4 ± 0.13	2.6 ± 0.22	3.0 ± 0.4	3.5 ± 0	1.16 ± 0.16
11	4.3 ± 0.21	3.6 ± 0.16	3.4 ± 0.16	2.6 ± 0.16	3.0 ± 0.2	3.5 ± 0.22	1.16 ± 0.16
12	4.6 ± 0.16	4.1 ± 0.23	3.9 ± 0.1	3.0 ± 0.29	3.16 ± 0.1	3.66 ± 0.21	1.0 ± 0.25

Fig. 1 illustrates the temporal profile of seizure severity scores across the 12 injection days (every other day over a 4-week period). Data are presented as mean ± S.E.M. (error bars). Normality of distribution was confirmed using the Kolmogorov–Smirnov test, and individual data points are shown to illustrate variability.

Treatment with topiramate alone reduced seizure scores in a dose- and time-dependent manner. Compared to the Seizure group, the TPM (25 mg) and TPM (50 mg) groups did not show statistically significant differences until the final days of the experiment. In contrast, the TPM (70 mg) group showed a significant reduction from day 9 onward, with the effect becoming more pronounced over time (day 9: 3.5 ± 0.22 vs. 2.3 ± 0.15, *p < 0.01*; day 12: 4.6 ± 0.16 vs. 3.0 ± 0.29, *p < 0.001*; n = 10 per group). The detailed daily data for all groups are presented in Table 1 and Fig. 1.

Exercise combined with topiramate also led to reductions in seizure scores. The EX + TPM (25 mg) group showed a significant reduction on days 11 (3.0 ± 0.2 vs. 4.3 ± 0.21, *p < 0.05*, n = 10) and 12 (3.16 ± 0.1 vs. 4.6 ± 0.16, *p < 0.05*, n = 10) relative to the Seizure group. The EX + TPM (70 mg) group exhibited a significant reduction relative to the Seizure group from day 6 onward, with the effect becoming more pronounced over time (day 6: 0.83 ± 0.3 vs. 2.4 ± 0.26, *p < 0.05*; day 12: 1.0 ± 0.25 vs. 4.6 ± 0.16, *p < 0.001*; n = 10 per group). The detailed daily data for all groups are presented in Table 1 and Fig. 1.

A direct comparison between the combination and monotherapy groups revealed specific differences. The EX + TPM (70 mg) group had significantly lower seizure scores than the TPM (70 mg) group from day 9 onward, with the effect becoming more pronounced over time (day 9: 1.16 ± 0.16 vs. 2.3 ± 0.15, *p < 0.05*; day 12: 1.0 ± 0.25 vs. 3.0 ± 0.29, *p < 0.001*; n = 10 per group). Furthermore, seizure scores in the EX + TPM (70 mg) group were significantly lower than those in the TPM (25 mg) and TPM (50 mg) groups from day 9 onward (*p < 0.001*, n = 10 per group; see Table 1 and Fig. 1 for daily data).

A dose-dependent effect was observed within both treatment strategies. In the monotherapy groups, seizure scores in the TPM (70 mg) group were significantly lower than those in the TPM (25 mg) group from day 9 through day 12 (*p < 0.05*, n = 10 per group; see Table 1 for daily data). Similarly, among the combination therapy groups, the EX + TPM (70 mg) group showed significantly lower seizure scores than the EX + TPM (25 mg) group from day 6 onward, and significantly lower scores than the EX + TPM (50 mg) group from day 9 onward (*p < 0.001*, n = 10 per group; see Table 1 for daily data).

### 3.2 Latency of the first seizure appearance

**Fig. 2:**
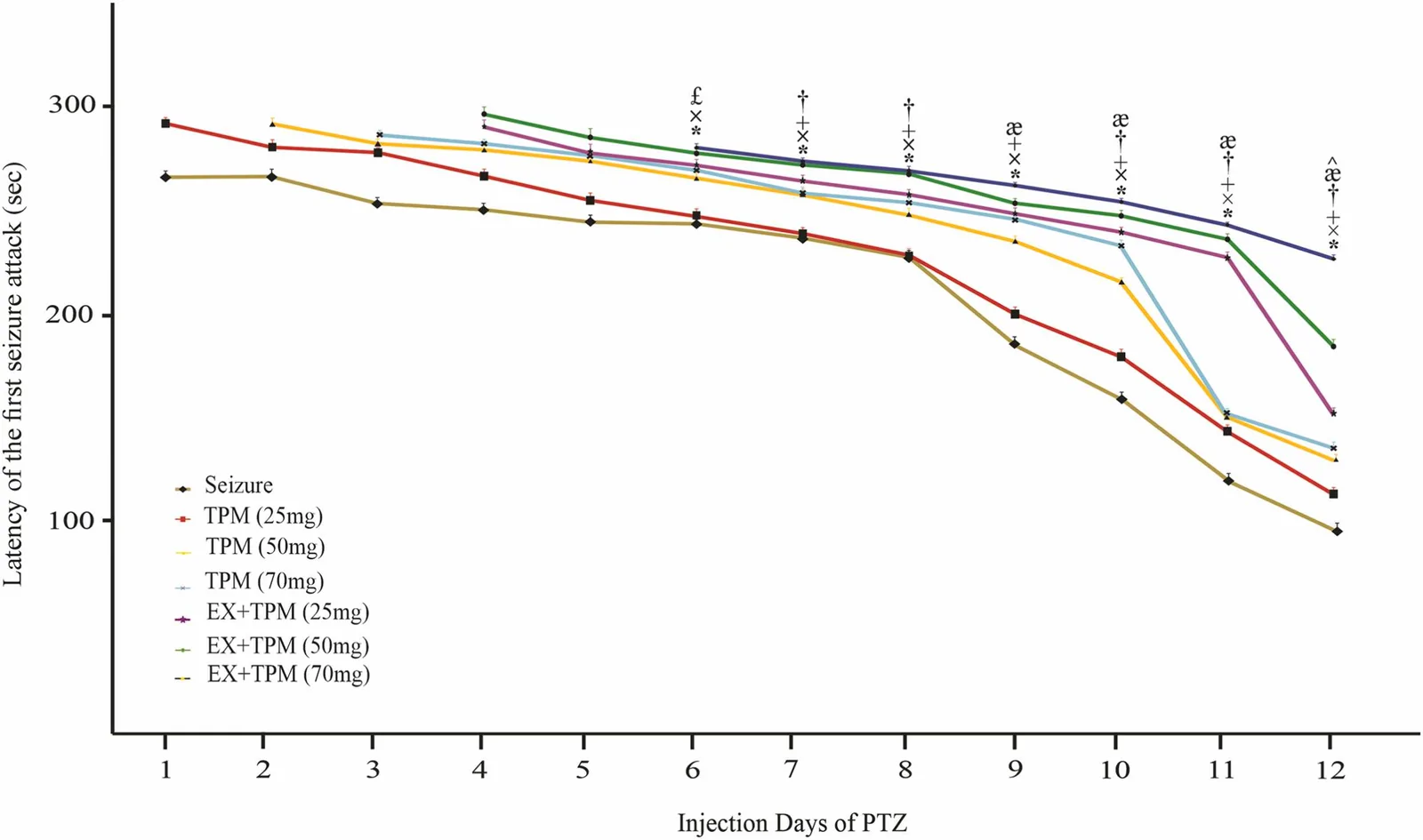
Effects of monotherapy and combination therapy on seizure latency The line graph shows the time latency to the first PTZ-induced seizure. Combination therapy with moderate-intensity treadmill and topiramate (EX+TPM groups) significantly prolonged the seizure latency compared with the Seizure group and their respective topiramate monotherapy groups (TPM) at all dose levels (25, 50, and 70 mg). The most potent effect was observed in the EX + TPM (70 mg) group. Data are presented as mean ± S.E.M. (error bars). Normality of distribution was confirmed using the Kolmogorov-Smirnov test. The symbols *, × , + , †, æ, ^, and £ indicate a significant difference in comparison with the Seizure, TPM (25 mg), TPM (50 mg), TPM (70 mg), EX + TPM (25 mg), EX + TPM (50 mg), and EX + TPM (70 mg) groups, respectively.

Latency to seizure onset (mean ± S.E.M., in seconds) is presented in Table 2 and Fig. 2.

**Table 2:** Mean ± S.E.M. of seizure latency.

Injections	Groups						
	Seizure	TPM (25 mg)	TPM (50 mg)	TPM (70 mg)	EX + TPM (25 mg)	EX + TPM (50 mg)	EX + TPM (70 mg)
1	270.14 ± 2.34	292.2 ± 2.28	0	0	0	0	0
2	269.85 ± 2.85	280.8 ± 3.05	292.0 ± 2.3	0	0	0	0
3	256.37 ± 1.6	287.3 ± 2.31	282.5 ± 2.39	285.55 ± 1.63	0	0	0
4	262.8 ± 2.71	267 ± 2.75	279.5 ± 3.27	281.66 ± 2.35	290.5 ± 3.13	297.0 ± 3.18	0
5	246.3 ± 2.67	255.4 ± 2.96	274.1 ± 2.53	274.2 ± 0.9	278.0 ± 3.72	285.83 ± 3.97	0
6	244.9 ± 1.95	247.7 ± 2.43	266.0 ± 3.66	269.6 ± 1.46	272.16 ± 1.93	278.33 ± 1.6	280.66 ± 1.22
7	237.5 ± 2.29	239.5 ± 1.68	257.9 ± 2.91	260.5 ± 2.46	264.66 ± 2.23	272.66 ± 1.62	274.16 ± 1.24
8	227.7 ± 2.81	229.2 ± 1.39	248.3 ± 2.43	253.4 ± 2.81	258.16 ± 2.13	268.5 ± 1.56	269.5 ± 1.33
9	184.7 ± 1.94	200.9 ± 2.24	235.7 ± 2.05	245.4 ± 2.03	249.0 ± 2.19	254.33 ± 1.05	262.5 ± 0.88
10	158.1 ± 2.06	180.4 ± 1.95	216.7 ± 1.37	233.4 ± 3.03	240.16 ± 2.35	248.33 ± 1.42	254.66 ± 1.08
11	118.3 ± 1.28	144.6 ± 2.68	151.9 ± 1.55	154.0 ± 0.81	228.0 ± 2.64	236.66 ± 1.49	243.5 ± 0.92
12	94.1 ± 2.24	114.5 ± 2.83	130.8 ± 2.31	136.7 ± 2.91	152.66 ± 2.12	185.33 ± 1.78	227.33 ± 1.96

Fig. 2 illustrates the time course of seizure latency across the 12 injection days. Data are presented as mean ± S.E.M. (error bars). Normality of distribution was confirmed using the Kolmogorov–Smirnov test.

No significant differences were observed between any groups during the first five injections.

From day 6 onward, all treatment groups showed significant increases in seizure latency compared to the Seizure group (*p < 0.001*). On day 6, the latencies for the TPM (50 mg), TPM (70 mg), EX + TPM (25 mg), EX + TPM (50 mg), and EX + TPM (70 mg) groups were 266.0 ± 3.66, 269.6 ± 1.46, 272.16 ± 1.93, 278.33 ± 1.6, and 280.66 ± 1.22 s, respectively, compared to 244.9 ± 1.95 s in the Seizure group. The effect became more pronounced over time, with the EX + TPM (70 mg) group reaching a latency of 227.33 ± 1.96 s on day 12, compared to 94.1 ± 2.24 s in the Seizure group (*p < 0.001*, n = 10 per group).

A dose-dependent increase in latency was observed among both monotherapy and combination therapy groups, with higher doses producing greater effects (*p < 0.001*, n = 10 per group). The combination therapy groups consistently showed longer latencies than their respective monotherapy groups. The detailed daily data for all groups are presented in Table 2 and Fig. 2.

### 3.3 Depression-like behaviors assessment by the Tail Suspension Test (TST)

**Fig. 3:**
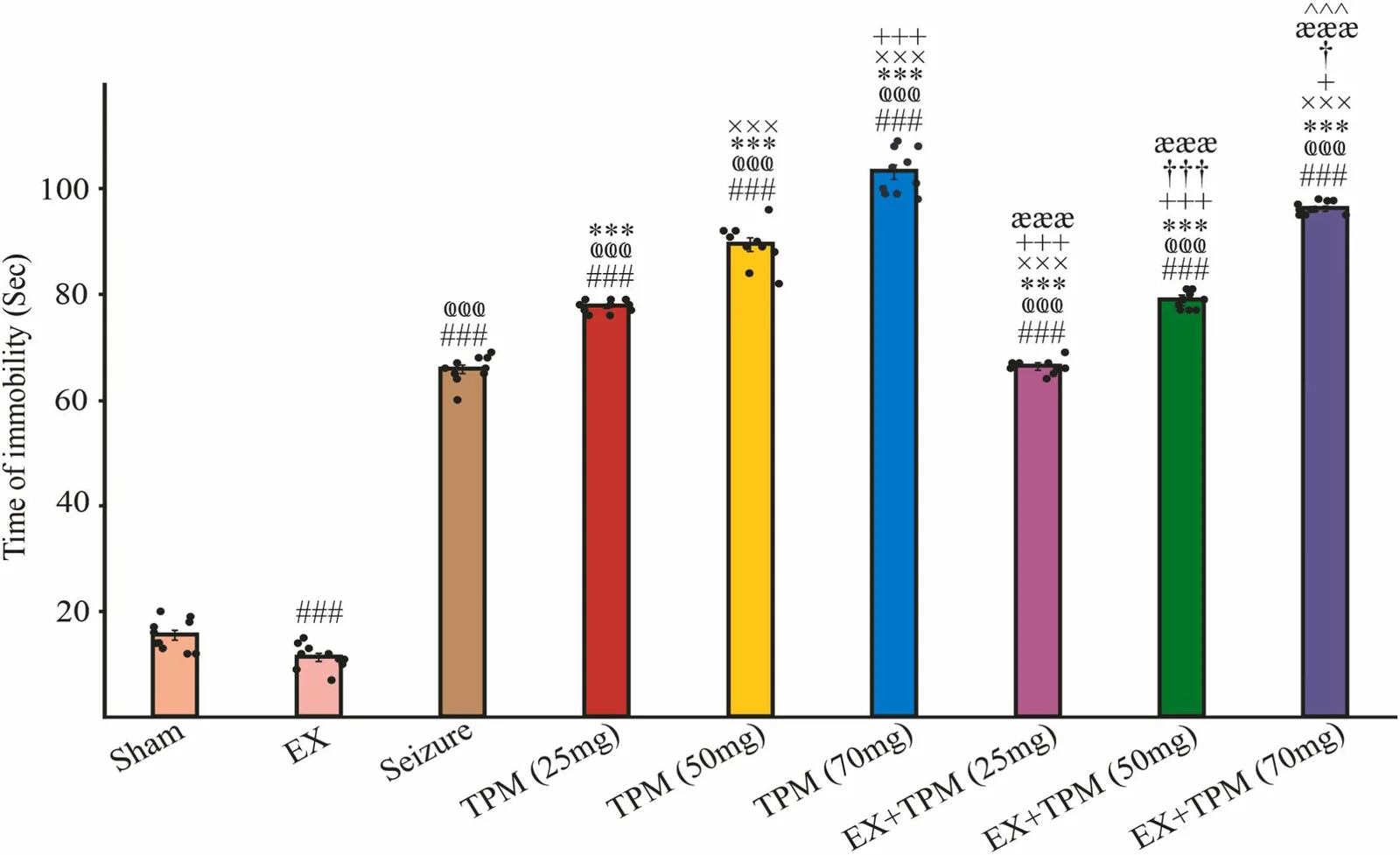
Immobility time in the Tail Suspension Test. Data on immobility time are presented as mean ± S.E.M. (error bars). Normality of distribution was confirmed using the Kolmogorov-Smirnovtest, and individual data points are shown to illustrate variability. The combination therapy of exercise and TPM significantly reduced immobility time compared to the monotherapy groups. The symbols #, Ҩ, *, × , + , †, æ, ^, and £ indicate a significant difference from Sham, EX, Seizure, TPM (25 mg), TPM (50 mg), TPM (70 mg), EX + TPM (25 mg), EX + TPM (50 mg), and EX + TPM (70 mg) groups respectively. One, two, and three repetitions of a symbol indicate *p < 0.05, p < 0.01,* and *p < 0.001*, respectively.

Immobility time during the Tail Suspension Test, a behavioral index of depressive-like state, is presented as mean ± S.E.M. in Table 3 and Fig. 3.

**Table 3:** Mean ± S.E.M. of immobility (Sec).

Sham	EX	Seizure
15.5 ± 0.92	11.3 ± 0.76	65.8 ± 0.81
TPM (25 mg)	**TPM (50 mg)**	**TPM (70 mg)**
77.7 ± 0.36	89.4 ± 1.29	103.1 ± 1.33
EX + TPM (25 mg)	**EX + TPM (50 mg)**	**EX + TPM (70 mg)**
66.33 ± 0.71	78.83 ± 1.01	96.16 ± 0.47

Fig. 3 illustrates the immobility time during the Tail Suspension Test across different experimental groups. Data are presented as mean ± S.E.M. (error bars). Normality of distribution was confirmed using the Kolmogorov–Smirnov test, and individual data points are shown to illustrate variability.

Animals in the Seizure group showed a significant increase in immobility time compared to the Sham group (65.8 ± 0.81 vs. 15.5 ± 0.92, *p < 0.001*, n = 10). All TPM monotherapy groups (25, 50, and 70 mg) also showed significantly longer immobility relative to the Sham group (77.7 ± 0.36, 89.4 ± 1.29, 103.1 ± 1.33 vs. 15.5 ± 0.92, *p < 0.001*, n = 10 for all groups).

Among the combination therapies, the EX + TPM (25 mg) and EX + TPM (50 mg) groups showed significantly lower immobility times compared to the Seizure group (66.33 ± 0.71 and 78.83 ± 1.01 vs. 65.8 ± 0.81, respectively), but both remained significantly higher than the Sham group (15.5 ± 0.92, *p < 0.001*, n = 10 for all comparisons). In contrast, the EX + TPM (70 mg) group exhibited a significant increase in immobility compared to the Sham group (96.16 ± 0.47 vs. 15.5 ± 0.92, *p < 0.001*, n = 10), while still showing a significant decrease relative to the Seizure group (96.16 ± 0.47 vs. 65.8 ± 0.81, *p < 0.001*).

The EX + TPM (25 mg) group showed a significant reduction in immobility compared with its corresponding monotherapy dose, TPM (25 mg), as well as compared with the higher-dose TPM (50 mg) and TPM (70 mg) groups (*p < 0.001*, n = 10 for all). Similarly, the EX + TPM (50 mg) and EX + TPM (70 mg) groups exhibited significantly shorter immobility than their respective monotherapy groups (*p < 0.001*, n = 10 for all). A comparison between the EX + TPM groups showed a dose-dependent effect; the EX + TPM (70 mg) group showed significantly longer immobility than both the EX + TPM (25 mg) and EX + TPM (50 mg) groups (*p < 0.001*, n = 10). The detailed data for all groups are presented in Table 3 and Fig. 3.

### 3.4 Effects of exercise and topiramate on anxiety-like behaviors

**Fig. 4:**
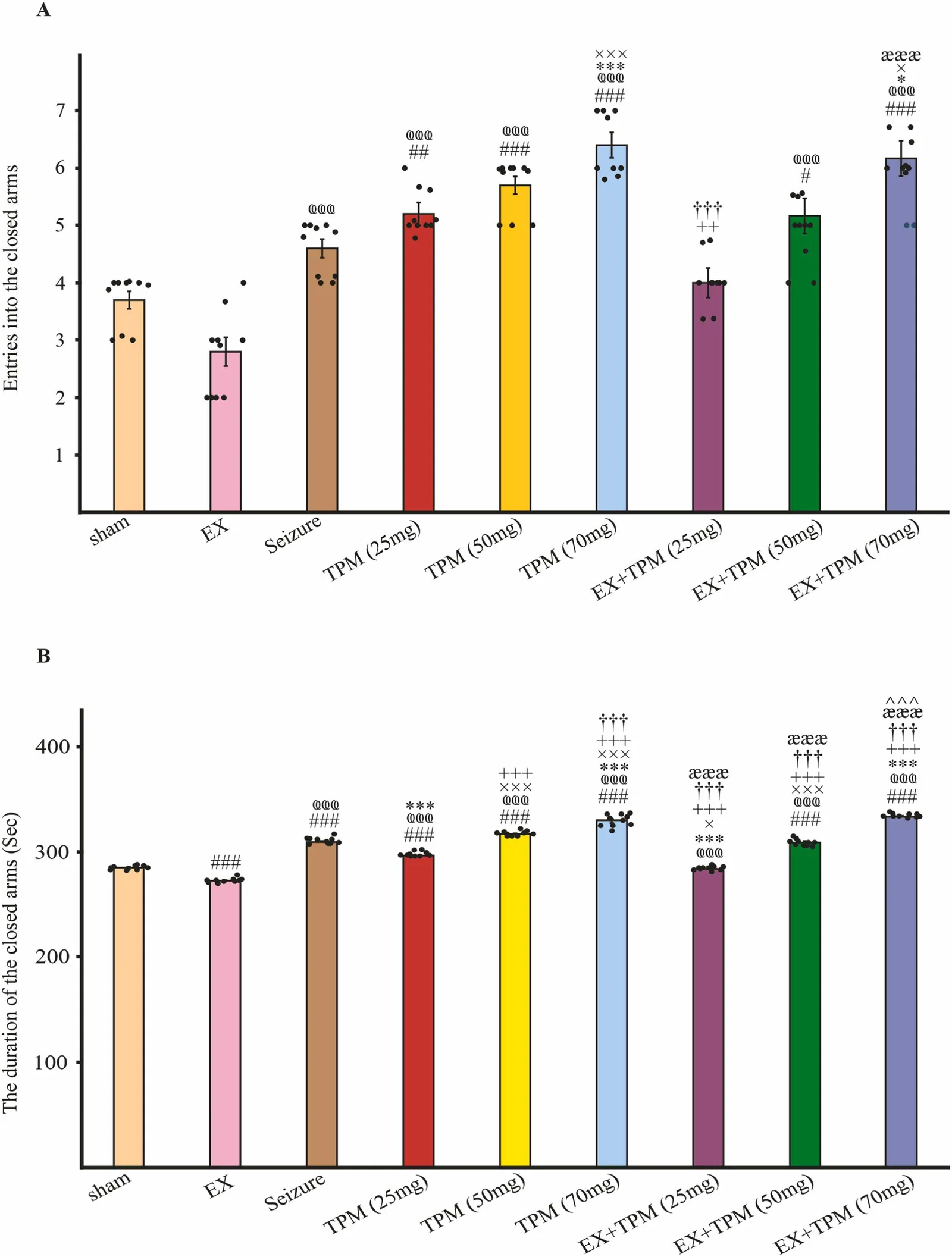
Assessment of anxiety-like behavior in the Elevated Plus Maze. **(A)** The mean number of entries into the closed arms is presented as mean ± S.E.M. (error bars). Normality of distribution was confirmed using the Kolmogorov-Smirnovtest, and individual data points are shown to illustrate variability. The observed reduction in closed-arm entries in the combination therapy groups suggests an attenuation of the depression-like behavioral phenotype associated with Seizure and topiramate monotherapy. **(B)** The mean of time spent in the closed arms is presented as mean ± S.E.M. (error bars). Normality of distribution was confirmed using the Kolmogorov-Smirnov test, and individual data points are shown to illustrate variability. Administration of topiramate (TPM) dose-dependently increased both parameters, an effect that was significantly counteracted by concurrent moderate-intensity treadmill exercise (EX+TPM), especially at higher doses. The symbols #, Ҩ, *, × , + , †, æ, ^, and £ indicate a significant difference in comparison with the Sham, EX, Seizure, TPM (25 mg), TPM (50 mg), TPM (70 mg), EX + TPM (25 mg), EX + TPM (50 mg), and EX + TPM (70 mg) groups respectively. One, two, or three repetitions of symbols indicate *p < 0.05*, *p < 0.01*, *and p < 0.001*, respectively*.*

The number of entries into and the time spent in the closed arms were measured. Data are presented as mean ± S.E.M. in Table 4, Table 5, and Fig. 4, respectively.

**Table 4:** Mean ± S.E.M. of entries into the closed arms.

Sham	EX	Seizure
3.7 ± 0.15	2.8 ± 0.13	4.6 ± 0.16
TPM (25 mg)	**TPM (50 mg)**	**TPM (70 mg)**
5.3 ± 0.15	5.7 ± 0.15	6.4 ± 0.16
EX + TPM (25 mg)	**EX + TPM (50 mg)**	**EX + TPM (70 mg)**
4.33 ± 0.21	5.16 ± 0.16	6.16 ± 0.16

**Table 5:** Mean ± S.E.M. of duration in the closed arms.

Sham	EX	Seizure
285.3 ± 0.53	272.6 ± 0.42	309.8 ± 0.46
TPM (25 mg)	**TPM (50 mg)**	**TPM (70 mg)**
296.7 ± 0.26	317.0 ± 0.55	329.3 ± 1.83
EX + TPM (25 mg)	**EX + TPM (50 mg)**	**EX + TPM (70 mg)**
284.66 ± 0.98	308.83 ± 1.4	333.83 ± 0.47

Fig. 4 illustrates the number of entries into and the time spent in the closed arms of the elevated plus maze across different experimental groups. Data are presented as mean ± S.E.M. (error bars). Normality of distribution was confirmed using the Kolmogorov–Smirnov test, and individual data points are shown to illustrate variability.

Animals in the Seizure group showed a significant increase in both the number of entries into (4.6 ± 0.16 vs. 3.7 ± 0.15, *p < 0.001*, n = 10) and time spent in (309.8 ± 0.46 vs. 285.3 ± 0.53, *p < 0.001*, n = 10) the closed arms compared with the Sham group. All TPM monotherapy groups also spent significantly more time in the closed arms compared with the Sham group (296.7 ± 0.26, 317 ± 0.55, 329.3 ± 1.83 for TPM 25, 50, and 70 mg, respectively; *p < 0.001*, n = 10).

Compared with the Seizure group, the TPM (50 mg) and TPM (70 mg) groups showed a significant increase in closed-arm time, while the TPM (25 mg) group showed a significant decrease (*p < 0.01*, n = 10). Similarly, the EX + TPM (25 mg) group showed a significant decrease in closed-arm time compared to the Seizure group, whereas the EX + TPM (70 mg) group showed a significant increase (*p < 0.01*, n = 10). No significant difference was observed between the Seizure and EX groups for either parameter.

Administration of TPM alone at all doses significantly increased the number of closed-arm entries compared with both the Sham and EX groups (*p < 0.001*, n = 10 for all). A dose-dependent effect was evident among monotherapy groups; the TPM (70 mg) group showed a significantly higher number of closed-arm entries than the TPM (25 mg) group (*p < 0.01*, n = 10).

The EX + TPM (25 mg) group showed a significantly lower number of closed-arm entries than the TPM (50 mg) and TPM (70 mg) groups (*p < 0.05* and *p < 0.001*, respectively, n = 10), while the EX + TPM (70 mg) group exhibited a significantly higher number of closed-arm entries than the TPM (50 mg) group (*p < 0.01*, n = 10). A dose-response relationship was maintained within the combination therapy; the EX + TPM (70 mg) group made a significantly higher number of entries than the EX + TPM (25 mg) group (*p < 0.001*, n = 10). The detailed data for all groups are presented in Table 4, Table 5, and Fig. 4.

As shown in Table 4, Table 5**,** and Fig. 4**,** the EPM revealed significant changes in anxiety-like behavior across groups. The TST, which specifically measures depressive-like behavior, showed that exercise combined with topiramate significantly reduced immobility time. Taken together, these results suggest that the combination therapy improved both anxiety-like and depressive-like behaviors in epileptic rats.

### 3.5 5-HT1A receptor immunoreactive staining intensity in the CA1 subfields of the hippocampus

**Fig. 5:**
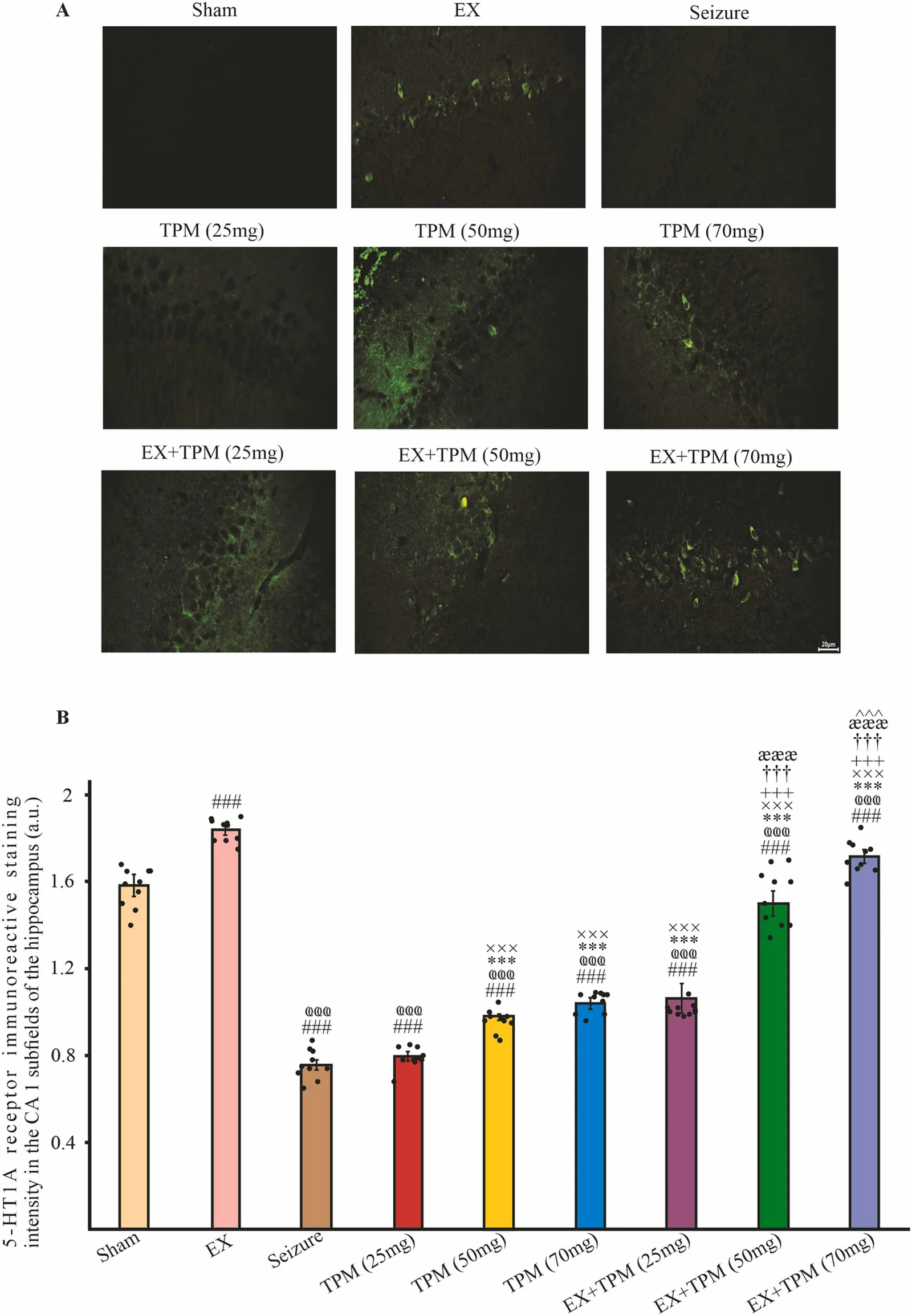
5-HT1A receptor immunoreactive staining intensity (Arbitrary Units, AU) in the CA1 subfields of the hippocampus are presented as mean ± S.E.M. (error bars). Normality of distribution was confirmed using the Kolmogorov-Smirnov test, and individual data points are shown to illustrate variability. **(A)** Immunofluorescent photomicrographs of 5-HT1A receptor expression (Arbitrary Units, AU) in the CA1 subfields of the hippocampus. **(B)** Bar charts summarize the mean 5-HT1A receptor immunoreactive staining intensity (Arbitrary Units, AU). The 5-HT1A receptor immunoreactive staining intensity (Arbitrary Units, AU) was significantly upregulated in the EX + TPM (50 mg) and EX + TPM (70 mg) groups in the CA1 subfields of the hippocampus. The symbols #, Ҩ, *, × , + , †, æ, ^, and £ indicate a significant difference in comparison with the Sham, EX, Seizure, TPM (25 mg), TPM (50 mg), TPM (70 mg), EX + TPM (25 mg), EX + TPM (50 mg), and EX + TPM (70 mg) groups respectively. One, two, or three repetitions of symbols indicate *p < 0.05*, *p < 0.01*, and *p < 0.001*, respectively.

Immunofluorescence assessment using the anti−5-HT1A receptor antibody was performed to quantify the immunoreactive staining intensity in the CA1 subfields of the hippocampus. The results are presented in Table 6 and Fig. 5 (A, B).

**Table 6:** Mean ± S.E.M. of 5-HT1A receptor immunoreactive staining intensity (Arbitrary Units, AU) in the CA1 subfields of the hippocampus.

Sham	EX	Seizure
1.58 ± 0.15	1.84 ± 0.02	0.75 ± 0.02
TPM (25 mg)	**TPM (50 mg)**	**TPM (70 mg)**
0.79 ± 0.02	0.97 ± 0.01	1.04 ± 0.02
EX + TPM (25 mg)	**EX + TPM (50 mg)**	**EX + TPM (70 mg)**
1.06 ± 0.06	1.5 ± 0.05	1.71 ± 0.03

Data are presented as mean ± S.E.M. (error bars). Normality of distribution was confirmed using the Kolmogorov–Smirnov test, and individual data points are shown to illustrate variability.

The immunoreactive staining intensity of 5-HT1A receptors in the EX group was significantly higher than the Sham group (1.84 ± 0.02 vs. 1.58 ± 0.15, *p < 0.001*, n = 10). In contrast, it was significantly lower in the Seizure group compared with both the Sham (0.75 ± 0.02 vs. 1.58 ± 0.15, *p < 0.001*, n = 10) and EX (0.75 ± 0.02 vs. 1.84 ± 0.02, *p < 0.001*, n = 10) groups.

Administration of TPM monotherapy significantly affected 5-HT1A receptor expression. All TPM groups (25, 50, and 70 mg) showed significantly lower staining intensity compared with the Sham group (0.79 ± 0.02, 0.97 ± 0.01, 1.04 ± 0.02 vs. 1.58 ± 0.15; *p < 0.05* to *p < 0.001*, n = 10). Similarly, these groups were significantly lower than the EX group (*p < 0.001* for TPM 25 mg and 50 mg; *p < 0.01* for TPM 70 mg).

Combination therapy yielded a distinct profile. The EX + TPM (25 mg) group showed no significant difference from the Sham group but significantly higher expression levels than the Seizure group (1.06 ± 0.06 vs. 0.75 ± 0.02, *p < 0.01*, n = 10). Both the EX + TPM (50 mg) and EX + TPM (70 mg) groups demonstrated no significant difference compared with the Sham and EX groups. Crucially, these two combination groups (EX+TPM 50 mg and 70 mg) showed significantly higher 5-HT1A receptor expression compared with the Seizure group (1.5 ± 0.05, 1.71 ± 0.03 vs. 0.75 ± 0.02; *p < 0.001* for both, n = 10).

Significantly higher 5-HT1A receptor expression was observed in all EX + TPM groups compared with their corresponding monotherapy doses (*p < 0.001* for EX+TPM (25 mg) vs. TPM (25 mg); *p < 0.01* for EX + TPM (50 mg) vs. TPM 50 mg and EX + TPM (70 mg) vs. TPM (70 mg); n = 10). Moreover, the EX + TPM (70 mg) group displayed significantly higher 5-HT1A receptor expression than both the EX + TPM (25 mg) (1.71 ± 0.03 vs. 1.06 ± 0.06, *p < 0.05*, n = 10) and the EX + TPM (50 mg) (1.71 ± 0.03 vs. 1.5 ± 0.05, *p < 0.05*, n = 10) groups. The detailed data for all groups are presented in Table 6 and Fig. 5.

### 3.6 5-HT1A receptor immunoreactive staining intensity in the CA3 subfields of the hippocampus

**Fig. 6:**
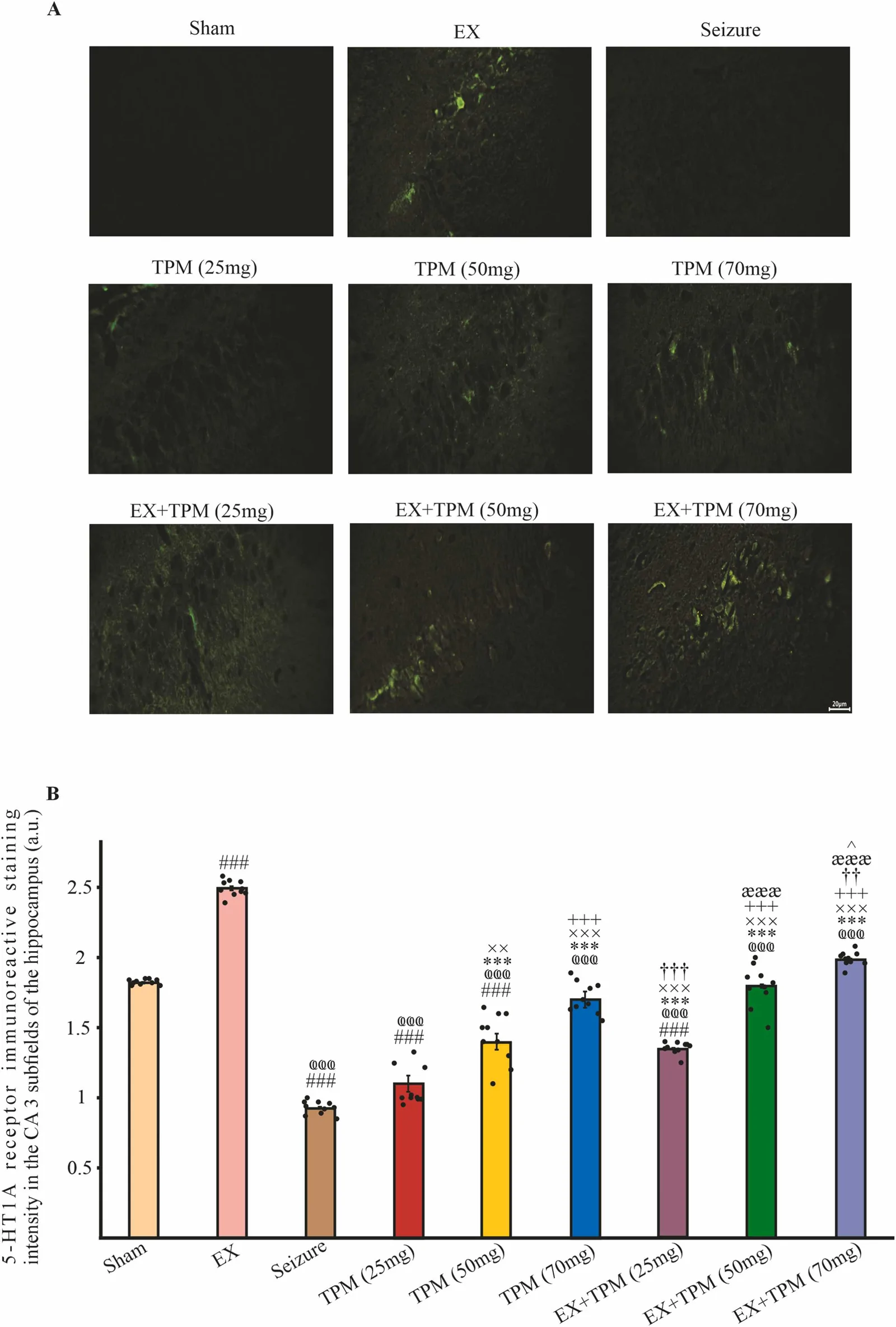
5-HT1A receptor immunoreactive staining intensity (Arbitrary Units, AU) in the CA3 subfields of the hippocampus is presented as mean ± S.E.M. (error bars). Normality of distribution was confirmed using the Kolmogorov-Smirnov test, and individual data points are shown to illustrate variability. **(A)** Immunofluorescent photomicrographs of 5-HT1A receptor expression (green) in the CA3. subfields of the hippocampus (Arbitrary Units, AU). **(B)** Bar charts summarize the mean 5-HT1A receptor immunoreactive staining intensity (Arbitrary Units, AU). The 5-HT1A receptor immunoreactive staining intensity (Arbitrary Units, AU) was significantly higher in the EX, TPM (50 mg), TPM (70 mg), EX + TPM (50 mg), and EX + TPM (70 mg) groups in the CA3 subfields of the hippocampus than in the other experimental The symbols #, Ҩ, *, × , + , †, æ, ^, and £ indicate a significant difference in comparison with the Sham, EX, Seizure, TPM (25 mg), TPM (50 mg), TPM (70 mg), EX + TPM (25 mg), EX + TPM (50 mg), and EX + TPM (70 mg) groups respectively. One, two, or three repetitions of symbols indicate *p < 0.05*, *p < 0.01*, and *p < 0.001*, respectively.

Effects of topiramate and exercise on 5-HT1A receptor expression in the CA3 subfields of the hippocampus, with full results, are depicted in Table 7 and Fig. 6 **(A, B)**.

**Table 7:** Mean ± S.E.M. of 5-HT1A receptor immunoreactive staining intensity (Arbitrary Units, AU) in the CA3 subfields of the hippocampus.

Sham	EX	Seizure
1.82 ± 0.005	2.49 ± 0.01	0.93 ± 0.005
TPM (25 mg)	**TPM (50 mg)**	**TPM (70 mg)**
1.1 ± 0.05	1.4 ± 0.05	1.7 ± 0.05
EX + TPM (25 mg)	**EX + TPM (50 mg)**	**EX + TPM (70 mg)**
1.35 ± 0.005	1.79 ± 0.01	1.98 ± 0.004

Data are presented as mean ± S.E.M. (error bars). Normality of distribution was confirmed using the Kolmogorov–Smirnov test, and individual data points are shown to illustrate variability.

The EX group showed a significantly higher 5-HT1A immunoreactive staining intensity compared with the Sham group (2.49 ± 0.01 vs. 1.82 ± 0.005, *p < 0.001*, n = 10). Conversely, the Seizure group displayed a significantly lower staining intensity relative to the Sham group (0.93 ± 0.005 vs. 1.82 ± 0.005, *p < 0.001*, n = 10). Moreover, the Seizure group showed significantly lower staining intensity than both the Sham (0.93 ± 0.005 vs. 1.82 ± 0.005, *p < 0.001*, n = 10) and the EX groups (0.93 ± 0.005 vs. 2.49 ± 0.01, *p < 0.001*, n = 10).

All TPM monotherapy groups had significantly lower 5-HT1A immunoreactive staining intensity compared with the Sham group (1.1 ± 0.05, 1.4 ± 0.05, 1.7 ± 0.05 vs. 1.82 ± 0.005; *p < 0.001* for all, n = 10). Similarly, they had significantly lower staining intensity than the EX group (*p < 0.001* for all, n = 10). When compared with the Seizure group, the TPM (25 mg) group showed no significant difference; however, both the TPM (50 mg) and TPM (70 mg) groups had significantly higher staining intensity relative to the Seizure group (1.4 ± 0.05, 1.7 ± 0.05 vs. 0.93 ± 0.005*; p < 0.001* for both, n = 10).

A dose-dependent effect was observed among monotherapy groups. The staining intensity in the TPM (70 mg) group was significantly higher than in the TPM (50 mg) group (1.7 ± 0.05 vs. 1.4 ± 0.05, *p < 0.01*, n = 10), which in turn was significantly higher compared with the TPM (25 mg) group (1.4 ± 0.05 vs. 1.1 ± 0.05, *p < 0.001*, n = 10).

The EX + TPM (25 mg) group showed a significantly lower staining intensity compared with the Sham group (1.35 ± 0.005 vs. 1.82 ± 0.005, *p < 0.001*, n = 10) and the EX group (1.35 ± 0.005 vs. 2.49 ± 0.01, *p < 0.001*, n = 10), but a significantly higher one compared with the Seizure group (1.35 ± 0.005 vs. 0.93 ± 0.005, *p < 0.001*, n = 10). In contrast, both the EX + TPM (50 mg) and EX + TPM (70 mg) groups exhibited higher staining intensity, which was not significantly different from that in the Sham group. While still lower than the EX group (1.79 ± 0.01, 1.98 ± 0.004 vs. 2.49 ± 0.01, *p < 0.001*, n = 10), these two combination groups showed significantly higher staining intensity relative to the Seizure group (1.79 ± 0.01, 1.98 ± 0.004 vs. 0.93 ± 0.005; *p < 0.001* for both, n = 10).

Combination therapy showed a superior effect compared to corresponding monotherapy doses. Both the EX + TPM (50 mg) and EX + TPM (70 mg) groups displayed significantly higher staining intensity than the TPM (50 mg) group (1.79 ± 0.01, 1.98 ± 0.004 vs. 1.4 ± 0.05; *p < 0.001* for both, n = 10). Crucially, the EX + TPM (70 mg) group showed a significantly higher staining intensity compared to the TPM (70 mg) group (1.98 ± 0.004 vs. 1.7 ± 0.05*, p < 0.01*, n = 10). Furthermore, the EX + TPM (70 mg) group displayed a significantly higher staining intensity than both the EX + TPM (25 mg) (1.98 ± 0.004 vs. 1.35 ± 0.005, *p < 0.001*, n = 10) and EX + TPM (50 mg) groups (1.98 ± 0.004 vs. 1.79 ± 0.01, *p < 0.01*, n = 10). The detailed data for all groups are presented in Table 7 and Fig. 6.

### 3.7 5-HT1A immunoreactive staining intensity in the cerebral cortex

**Fig. 7:**
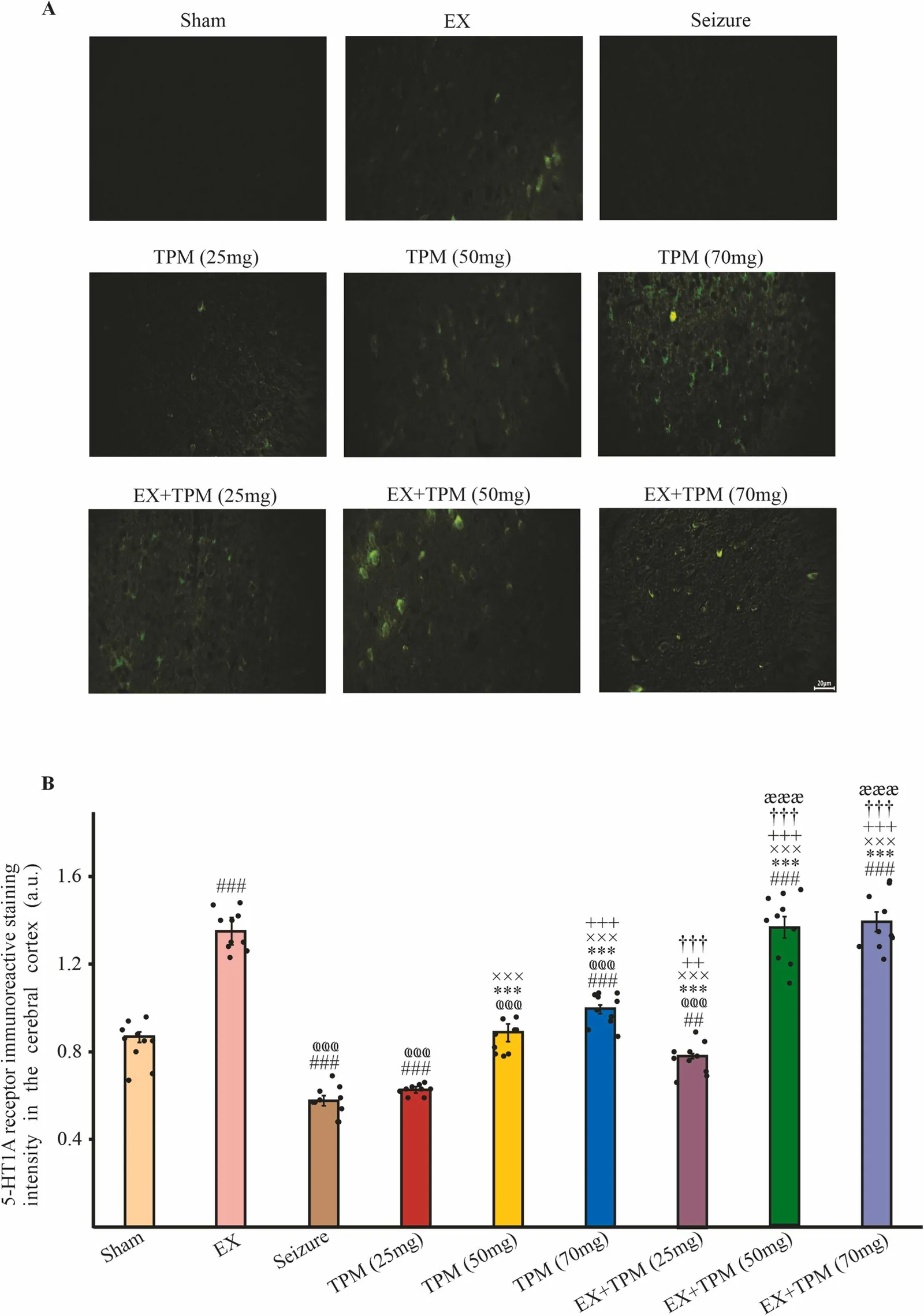
5-HT1A receptor immunoreactive staining intensity (Arbitrary Units, AU) in the cerebral cortex is presented as mean ± S.E.M. (error bars). Normality of distribution was confirmed using the Kolmogorov-Smirnov test, and individual data points are shown to illustrate variability. **(A)** Immunofluorescent photomicrographs of 5-HT1A receptor expression (Arbitrary Units, AU) (green) in the cerebral cortex. **(B)** Bar charts summarize the mean 5-HT1A receptor immunoreactive staining intensity (Arbitrary Units, AU). The 5-HT1A receptor immunoreactive staining intensity (Arbitrary Units, AU) was significantly higher in the EX, EX + TPM (50 mg), and EX + TPM (70 mg) groups in the cerebral cortex than in the other experimental groups. The symbols #, Ҩ, *, × , + , †, æ, ^, and £ indicate a significant difference in comparison with the Sham, EX, Seizure, TPM (25 mg), TPM (50 mg), TPM (70 mg), EX + TPM (25 mg), EX + TPM (50 mg), and EX + TPM (70 mg) groups respectively. One, two, or three repetitions of symbols indicate *p < 0.05*, *p < 0.01*, and *p < 0.001*, respectively.

Analysis of 5-HT1A receptor expression in the cerebral cortex with immunofluorescence revealed the significant modulatory effects of both EX and TPM treatments. Results are presented in Table 8 and Fig. 7 **(A, B)**.

**Table 8:** Mean ± S.E.M. of 5-HT1A receptor immunoreactive staining intensity (Arbitrary Units, AU) in the cerebral cortex.

Sham	EX	Seizure
0.86 ± 0.02	1.35 ± 0.06	0.57 ± 0.02
TPM (25 mg)	**TPM (50 mg)**	**TPM (70 mg)**
0.62 ± 0.01	0.88 ± 0.04	0.99 ± 0.02
EX + TPM (25 mg)	**EX + TPM (50 mg)**	**EX + TPM (70 mg)**
0.78 ± 0.01	1.36 ± 0.08	1.39 ± 0.15

Data are presented as mean ± S.E.M. (error bars). Normality of distribution was confirmed using the Kolmogorov–Smirnov test, and individual data points are shown to illustrate variability.

The EX group had a significantly higher 5-HT1A receptor expression level compared to the Sham group (1.35 ± 0.06 vs. 0.86 ± 0.02, *p < 0.05*, n = 10). In contrast, the Seizure group had a significantly lower expression level compared to the Sham group (0.57 ± 0.02 vs. 0.86 ± 0.02*, p < 0.01*, n = 10). The Seizure group had a significantly lower expression level than both the Sham (0.57 ± 0.02 vs. 0.86 ± 0.02, *p < 0.01*, n = 10) and the EX groups (0.57 ± 0.02 vs. 1.35 ± 0.06, *p < 0.001*, n = 10).

The TPM (25 mg) and TPM (50 mg) monotherapy groups had a significantly lower expression level compared to the Sham group (0.62 ± 0.01, 0.88 ± 0.04 vs. 0.86 ± 0.02; *p < 0.05* for both, n = 10). The TPM (70 mg) group showed no significant difference from the Sham group. All TPM monotherapy groups had a significantly lower expression level than the EX group (*p < 0.001* for TPM (25 mg); *p < 0.05* for TPM (50 mg); *p < 0.01* for TPM (70 mg), n = 10). Compared with the Seizure group, the TPM (25 mg) group showed no significant difference. In comparison, the TPM (50 mg) group and the TPM (70 mg) group had higher expression levels (0.57 ± 0.02 vs. 0.88 ± 0.04, *p < 0.05*, and 0.57 ± 0.02 vs. 0.99 ± 0.02, *p < 0.01*, respectively).

A dose-dependent trend was observed. The TPM (70 mg) group (0.99 ± 0.02) had a significantly higher expression level than both the TPM (25 mg) (0.62 ± 0.01) and TPM (50 mg) (0.88 ± 0.04) groups (*p < 0.05* for both comparisons, n = 10).

The EX + TPM (25 mg) group showed no significant difference from the Sham group but had a significantly lower expression level than the EX group (0.78 ± 0.01 vs. 1.35 ± 0.06, *p < 0.01*, n = 10). Both the EX + TPM (50 mg) and EX + TPM (70 mg) groups had a significantly higher expression level compared to the Sham group (1.36 ± 0.08, 1.39 ± 0.15 vs. 0.86 ± 0.02; *p < 0.01* for both, n = 10) and the Seizure group (0.57 ± 0.02; *p < 0.001* for both, n = 10); however, they had a significantly lower expression level than the EX group (1.35 ± 0.06; *p < 0.05* for both, n = 10).

Combination therapy demonstrated superior efficacy. The EX + TPM (50 mg) and EX + TPM (70 mg) groups had a significantly higher expression level than the TPM (25 mg) group (1.36 ± 0.08, 1.39 ± 0.15 vs. 0.62 ± 0.01; *p < 0.001* for both, n = 10), as did the EX + TPM (50 mg) group compared with the TPM (50 mg) group (1.36 ± 0.08 vs. 0.88 ± 0.04, *p < 0.05*, n = 10). Moreover, the EX + TPM (70 mg) group had a significantly higher expression level compared with both the TPM (50 mg) (1.39 ± 0.15 vs. 0.88 ± 0.04, *p < 0.01*, n = 10) and TPM (70 mg) groups (1.39 ± 0.15 vs. 0.99 ± 0.02, *p < 0.05*, n = 10).

A clear dose-response effect was evident with combination therapy. Both the EX + TPM (50 mg) and EX + TPM (70 mg) groups had significantly higher expression levels than the EX + TPM (25 mg) group (1.36 ± 0.08, 1.39 ± 0.15 vs. 0.78 ± 0.01; *p < 0.001* for all, n = 10). The EX + TPM (70 mg) group also had a significantly higher expression level compared with the EX + TPM (50 mg) group (1.39 ± 0.15 vs. 1.36 ± 0.08, *p < 0.01*, n = 10). The detailed data for all groups are presented in Table 8 and Fig. 7.

## 4 Discussion

The integration of non-pharmacological interventions with established pharmacotherapies represented a promising frontier in the holistic management of neurological disorders. This study provided compelling preclinical evidence that moderate-intensity treadmill exercise synergistically enhanced the therapeutic profile of topiramate in a chronic model of PTZ-kindling. This synergy manifested not only as a potentiation of antiseizure efficacy but, more notably, as an attenuation of the drug's adverse depressive-like effects. These behavioral outcomes were associated with a concomitant upregulation of 5-HT1A receptor expression level in key brain regions, offering a plausible neurobiological substrate for the observed benefits.

### 4.1 Combination therapy of topiramate and exercise improved the dose-efficacy profile

Our findings delineate a clear superiority of the combination regimen over monotherapy in controlling convulsive activity. While topiramate alone produced the expected dose-dependent reduction in seizure scores and prolongation of seizure latency, the temporal kinetics and magnitude of these effects were markedly amplified by concurrent exercise. Notably, the high-dose combination group (EX+TPM (70 mg)) exhibited a significant reduction in seizure scores from day 6. In contrast, the corresponding monotherapy group (TPM (70 mg)) achieved a comparable effect only from day 9 onward. Moreover, direct comparisons revealed that seizure scores in the EX + TPM (70 mg) group were significantly lower than those in the TPM (70 mg) group at the experiment's endpoint. These findings suggest that exercise fundamentally altered the brain's responsiveness to topiramate.

We posit that this pharmacodynamic synergy arises from complementary mechanisms. Voltage-gated sodium channel blockade (Zona et al., 1997), enhancement of GABAergic neurotransmission, and reduction of AMPA/kainate glutamate receptor activation (Skradski and White, 2000) are involved in the antiseizure effects of topiramate. Exercise induces broad-spectrum neuroplastic and homeostatic adaptations, including upregulation of neurotrophic factors such as BDNF (Mozhgan Abdullahzadeh Nobejari et al., 2024), enhancement of neuronal survival, and synaptic resilience (Mandolesi et al., 2018). It bolsters endogenous antioxidant defenses, reduces oxidative stress, and improves cerebral hemodynamics (Ebrahimnezhad et al., 2023). Critically, the present study identified upregulation of 5-HT1A receptor expression level in the hippocampal and cerebral cortex following combination therapy. The 5-HT1A receptor, an inhibitory G-protein-coupled receptor, induced neuronal hyperpolarization via GIRK channels (Ambrogini et al., 2023). This effect is mediated not only through direct postsynaptic inhibition but also through broader serotonergic modulation of network excitability. For instance, serotonin can indirectly control neuronal firing by facilitating GABAergic inhibition through mechanisms such as increased GABA release through interneuron activation (Bennett, 2026) and by dampening glutamatergic excitation through presynaptic inhibition of glutamate release (Nishijo et al., 2022). Its enhanced expression, likely driven by exercise, therefore provided a pervasive inhibitory and stabilizing tone across neural circuits through these multifaceted mechanisms, effectively raising the seizure threshold. Thus, when topiramate was administered, it acted upon a brain already primed for stability by exercise, resulting in a supra-additive antiseizure effect. This mechanistic overlap provided a rational basis for the observed dose-efficacy enhancement, implying that lower doses of topiramate may achieve therapeutic control when paired with exercise, thereby minimizing dose-dependent side effects.

### 4.2 Combination therapy of topiramate and exercise ameliorated drug-induced depressive-like behaviors

The behavioral findings of the current study demonstrate a clear mitigation of depressive-like phenotypes by combination therapy, in contrast to literature reports that high-dose topiramate monotherapy can exacerbate such behaviors (Lin et al., 2023, Phabphal and Udomratn, 2010). This study has shown that the PTZ-kindling process downregulated 5-HT1A receptor expression level, a pathophysiological deficit strongly associated with the emergence of depression in epilepsy. Critically, the combined intervention of exercise and topiramate was associated with the prevention of this receptor downregulation. This finding aligned with strong pharmacological evidence demonstrating that selective agonists of this receptor exert potent anticonvulsant effects across diverse animal models (López-Meraz et al., 2005).

The observed upregulation of 5-HT1A receptor expression level following combination therapy may have contributed to the improvement in depressive-like behaviors, consistent with the established role of this receptor in mood regulation. However, the precise intracellular signaling pathways underlying this effect were not examined in the present study and warrant further investigation.

Furthermore, the functional significance of 5-HT1A receptor upregulation in the hippocampus, as observed in the current study, is strongly supported by circuit-level evidence. Specifically, the integrity of the serotonergic projection from the dorsal raphe nucleus (DRN) to the hippocampus is fundamental for regulating mood and cognition. The compromised function of this specific DRN–hippocampal circuit has been directly and causally linked to the manifestation of both depressive and cognitive impairments in preclinical models (Chen et al., 2024). Therefore, the current finding that combination therapy was associated with increased hippocampal 5-HT1A receptor expression level suggests a potential restoration of signaling within this critical mood-regulating pathway, which may serve as a neurobiological correlate of the observed antidepressant effect.

The present study revealed a biphasic, dose-dependent effect of topiramate on anxiety-like behavior in the elevated plus maze (EPM). While the lowest dose (25 mg) reduced closed-arm time, suggesting an anxiolytic-like effect, higher doses (50 and 70 mg) significantly increased this parameter, indicating an anxiogenic response. This pattern was consistent with the complex pharmacological profile of topiramate. At lower doses, topiramate has been reported to exert anxiolytic-like effects in rodent models, an effect attributed to its modulatory actions on GABAergic and glutamatergic systems (Junqueira-Ayres et al., 2017). Mechanistically, this might be explained by the selective antagonism of GluR5 kainate receptors at low concentrations (Gryder and Rogawski, 2003), which reduced excitatory drive in the amygdala, a key region for anxiety regulation. In contrast, the emergence of anxiety at higher doses aligned with the well-documented, dose-dependent psychiatric side effects of topiramate, including anxiety and mood disturbances, as reported in preclinical and clinical trials and summarized in the FDA prescribing information (Physicians' Desk Reference, 2009, Kanner et al., 2003). This shift from an anxiolytic to an anxiogenic response likely reflected a change in the predominant mechanism of action as dosage increases, potentially involving broader modulation of AMPA receptors at higher concentrations (Gryder and Rogawski, 2003).These findings highlighted that the behavioral effects of topiramate were critically dose dependent.

Taken together, these findings suggested that the combination of exercise and topiramate exerts beneficial effects on mood-related behaviors, potentially through modulation of 5-HT1A receptor expression. However, further studies are needed to elucidate the precise mechanisms underlying these effects.

### 4.3 Assessment of emotional behaviors

In the present study, the elevated plus maze (EPM) and the tail suspension test (TST) were used to evaluate anxiety- and depressive-like behaviors, which frequently occur together in epilepsy patients and animal models.

The finding that exercise improved both anxiety-like (EPM) and depressive-like (TST) behaviors suggested a broad beneficial effect on mood, which is clinically relevant given the high comorbidity of anxiety and depression in epilepsy. However, we observed a significant association between exercise-induced 5-HT1A receptor upregulation and behavioral improvements, this was not establish causation. Future studies using 5-HT1A antagonists or genetic knockdown approaches wouhl be needed to determine causlity. We also recommend that future studies use additional validated tests, such as the forced swim test or sucrose preference test, to specifically assess depressive-like behavior.

## 5 Conclusion

In conclusion, this study demonstrated that moderate-intensity treadmill exercise enhanced the anticonvulsant efficacy of topiramate and attenuated its anxiety and depressive-like side effects in a rat model of epilepsy. These effects were associated with upregulation of 5-HT1A receptor expression level in the hippocampus and cerebral cortex. While these preclinical findings were promising and provided a strong rationale for further investigation, future translational and clinical studies were warranted before any clinical recommendations can be made.

## CRediT authorship contribution statement

**Fariba Karimzadeh:** Writing – review & editing, Validation, Supervision, Project administration, Methodology, Conceptualization. **Gelareh Vahabzadeh:** Investigation. **Homa Rasoolijazi:** Writing – review & editing, Validation, Supervision, Project administration, Conceptualization. **Babak Aliyari:** Investigation. **Mansoureh Soleimani:** Writing – review & editing, Visualization, Supervision, Project administration, Methodology, Conceptualization. **Ghazaleh Goudarzi:** Writing – original draft, Visualization, Software, Methodology, Investigation, Data curation.

## Compliance with ethical standards

This study was approved by the Ethics Committee of Iran University of Medical Sciences, Tehran, Iran (approval number: IR.IUMS.AEC.1402.111).

## Declaration of Generative AI and AI-assisted technologies in the writing process

During the preparation of this work, the authors used Deepseek to improve language and readability.

## Funding

This work was supported by a grant from Iran University of Medical Sciences, Tehran, Iran.

## Declaration of Competing Interest

None.
